# Iceland is an episodic source of atmospheric ice-nucleating particles relevant for mixed-phase clouds

**DOI:** 10.1126/sciadv.aba8137

**Published:** 2020-06-24

**Authors:** A. Sanchez-Marroquin, O. Arnalds, K. J. Baustian-Dorsi, J. Browse, P. Dagsson-Waldhauserova, A. D. Harrison, E. C. Maters, K. J. Pringle, J. Vergara-Temprado, I. T. Burke, J. B. McQuaid, K. S. Carslaw, B. J. Murray

**Affiliations:** 1 School of Earth and Environment, University of Leeds, Woodhouse Lane, Leeds LS2 9JT, UK.; 2 Agricultural University of Iceland, Hvanneyrabraut, 311 Hvanneyri, Iceland.; 3 Bison Engineering Inc., Helena, MT 59601, USA.; 4 Center for Geography and Environmental Science, University of Exeter, Penryn Campus, Penryn, Cornwall TR10 8FE, UK.; 5 Faculty of Environmental Sciences, Czech University of Life Sciences Prague, Kamycka 126, Prague 6, 16000, Czech Republic.; 6 Department of Chemistry, University of Cambridge, The Old Schools, Trinity Lane, Cambridge CB2 1TN, UK.; 7 Institute for Atmospheric and Climate Science, ETH Zurich, Zurich, Switzerland.

## Abstract

Ice-nucleating particles (INPs) have the potential to remove much of the liquid water in climatically important mid- to high-latitude shallow supercooled clouds, markedly reducing their albedo. The INP sources at these latitudes are very poorly defined, but it is known that there are substantial dust sources across the high latitudes, such as Iceland. Here, we show that Icelandic dust emissions are sporadically an important source of INPs at mid to high latitudes by combining ice-nucleating active site density measurements of aircraft-collected Icelandic dust samples with a global aerosol model. Because Iceland is only one of many high-latitude dust sources, we anticipate that the combined effect of all these sources may strongly contribute to the INP population in the mid- and high-latitude northern hemisphere. This is important because these emissions are directly relevant for the cloud-phase climate feedback and because high-latitude dust emissions are expected to increase in a warmer climate.

## Teaser

We show that Iceland contributes episodically to the regional atmospheric ice-nucleating particle population.

## INTRODUCTION

Atmospheric particles capable of nucleating ice can markedly alter the radiative properties of cold clouds. These particles are called ice-nucleating particles (INPs), and they can trigger heterogeneous ice formation in supercooled cloud droplets at temperatures well above those required for homogeneous ice nucleation (*1*). Droplet freezing triggers microphysical processes that can deplete supercooled liquid water and reduce cloud reflectivity, whereas the absence of primary ice production can lead to the persistence of supercooled liquid clouds (*2*). Hence, the abundance and activity of INPs are very important for cloud properties. Characterizing atmospheric INP concentrations is challenging in part because only a small subset of aerosol particles act as INP, and there are many particle types that can serve as INP. However, dust is thought to be one of the most important INP species around the globe, because of its ice-nucleating ability and its abundance (*3*–*6*).

Most of the dust in the Earth’s atmosphere is emitted by low-latitude arid and semi-arid sources such as the Sahara or Gobi Desert; hence, most of the dust transport research has focused on these low-latitude dust (LLD) sources (*7*, *8*). However, it is increasingly recognized that a substantial amount of dust is also emitted from high-latitude cold environments, such as proglacial deposits, contributing about 1 to 5% of the global dust budget (*9*–*12*). In addition, these dust sources have the potential to play an important role on a regional or even global scale (*10*, *13*–*17*). Furthermore, climate change may lead to decreased ice surface or snow cover, increasing emissions of high-latitude dust (HLD) in the future (*9*). It is therefore important to determine the ice-nucleating ability of HLDs and assess their source strength to establish how important they are for determining cloud glaciation. Recently, it was shown that dust particles derived from glacial Svalbard outwash plains are effective at nucleating ice (probably because of the presence of biological material) and might be an important source of atmospheric INPs at high latitudes (*11*).

In contrast to low-latitude sources, dust from high-latitude sources is emitted in a region of the world where they can directly affect the radiative properties of boundary layer mixed-phase clouds in a range of environments (*2*, *18*, *19*). In clouds at very high latitudes over sea ice and also in clouds over the Greenland ice sheet, HLD may affect the radiative energy budget of clouds, which are intricately linked to the local climate and are therefore important for sea ice loss (*20*) and ice sheet melt events (*21*). At mid to high latitudes over the open ocean, HLDs may play a critical role in the planet’s climate by reducing the liquid water path and albedo of shallow mixed-phase clouds (*2*). It is particularly important to accurately represent ice formation in these mixed-phase clouds because in a warmer future world models suggest that if INP concentrations remains constant, they will contain more liquid water and therefore become more reflective, with a substantial impact on equilibrium climate sensitivity (*22*). In addition, INP concentrations may change in a warmer world, with an impact on cloud phase. However, this cloud-phase feedback is highly uncertain at present, in part because of our lack of understanding of high-latitude INP sources in the present and future climate.

Iceland is an important HLD source, exporting dust to the atmosphere of the North Atlantic region throughout the year and therefore has the potential to contribute to the atmospheric INP burden (*9*, *14*, *16*). Icelandic dust has a distinct mineralogy compared to other HLDs. With volcanic eruptions happening on average every 3 to 5 years, the surface soils of Iceland are made from predominantly basaltic tephra and lava parent material, which has been chemically and physically weathered (e.g., by glacio-fluvial processes) (*23*). Common constituents of these soils include volcanic glass poor in silica and rich in metals, primary aluminosilicate (e.g., plagioclase, pyroxene, and olivine) and iron(-titanium) oxide (e.g., magnetite) minerals, and secondary minerals of varying crystallinity (e.g., allophane and ferrihydrite). These volcanic soils in “sandy deserts” across Iceland are susceptible to aerosolization through the action of wind, which produces frequent dust events (*9*, *14*–*17*, *24*). Furthermore, Icelandic dust can be transported to locations thousands of kilometers away from the original source (*25*) and can reach altitudes and latitudes where mixed-phase cloud formation can occur (*16*, *26*).

Very few studies have evaluated the ice-nucleating ability of Icelandic dust. A recent study showed that the ice nucleation activity of Icelandic glaciogenic silt at temperatures below −30°C was similar to that of dusts from low-latitude sources (*27*); no measurements have been made under conditions pertinent to most of the mixed-phase cloud systems in this region (i.e., above −30°C). The ice nucleation ability of volcanic ash from the 2010 eruption of Eyjafjallajökull volcano has also been studied and shown to be of a comparable activity to desert dust under mixed-phase conditions (*28*, *29*), while volcanic ash samples from other parts of the world show a great deal of variability in their activity (*30*, *31*). Given that dust from Iceland’s volcaniclastic deserts is processed through glacio-fluvial processes, its ice-nucleating activity may differ from freshly produced volcanic ash. In addition, Icelandic dust could contain ice-nucleating biogenic material, similarly to other HLDs (*11*).

Here, we characterize the immersion mode ice nucleation abilities of airborne-collected HLD samples under conditions pertinent to mixed-phase clouds using a droplet freezing assay to quantify atmospheric INP concentrations and scanning electron microscopy with energy-dispersive x-ray spectroscopy (SEM-EDS) to derive dust surface area. We then use the resulting temperature-dependent ice-active site density in combination with a global aerosol model, in which we have included an Icelandic dust emissions inventory to test how important Icelandic dust is as an INP source relative to LLD.

## RESULTS

### The ice-nucleating ability of airborne Icelandic dust

**Fig. 1:**
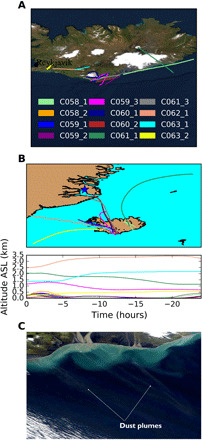
Sampling locations and air mass origins. (**A**) Flight track plots showing the location of the sampling of each pair of filters. The sampling altitudes varied from 30 to 2500 m. (**B**) The 24-hour Hybrid Single-Particle Lagrangian Integrated Trajectory (HYSPLIT) back trajectories of the air masses where each sample was collected. The blue star corresponds to a source of HLD in the east coast of Greenland that has been identified using NASA Worldview (e.g., 29 September 2018). The end points of the back trajectories are the mid-point of each filter sampling run. ASL, above sea level. (**C**) NASA Worldview satellite image of the south coast of Iceland on 2 October 2017, when the C058 and C059 flights were carried out. One can see dust plumes emanating from the south of Iceland, very close to the sampling locations.

To investigate the ice-nucleating ability of Icelandic dust, we measured both the concentration of INPs and the corresponding surface area of airborne Icelandic dust to derive the active sites per unit surface area (active site density). To do this, we sampled atmospheric aerosol particles using the filter inlet system on board of the United Kingdom’s BAe-146-301 Atmospheric Research Aircraft (managed by the FAAM Airborne Laboratory) during the VANAHEIM-2 campaign over the south of Iceland in October 2017. The filter inlet system can be used to sample aerosol particles smaller than ~20-μm particles on top of two filters simultaneously and has been characterized in a previous work (*32*). This allowed us to determine both the INP concentration using droplet freezing assays and the size-resolved composition of the aerosol particles by SEM-EDS on samples that were collected concurrently. The sampling flight tracks are shown in Fig. 1A. Further details of the samples are given in table S1.

To determine the surface area of dust particles on the filters, the size-resolved composition was obtained by SEM-EDS (*32*). This allowed us to obtain a direct size distribution of the aerosol particles on top of polycarbonate filters, as well as their size-resolved composition (see section S2). Size distributions from SEM-EDS were compared with those produced by the under-wing optical probes, and sampling biases in qualitative agreement with those reported previously (*32*) were observed. The surface areas of particles showing a chemical composition consistent with mineral dust or volcanic material (SEM-EDS categories: Al-Si rich, Si only, Si rich, Ca rich, and Metal rich) were calculated using an approach defined previously (*32*). Most of the analyzed samples (8 of 11) had a coarse surface area size distribution dominated by dust particles (comprising 88 to 99% of the total surface area), with a single mode centered between ~3 and ~8 μm. Some of the samples (the remaining 3 of 11) did not have dust concentrations substantially above the limit of detection (see section S2).

Dust from low-latitude sources as well as other high-latitude sources may be present in the air around Iceland; hence, we examined back trajectories and the chemical composition of the dust to determine its most likely source. Back-trajectory analysis (24-hour trajectories are shown in Fig. 1B) revealed that the air masses associated with almost all the samples dominated by coarse-mode dust had passed through the boundary layer (assuming to be 1 km deep) above the dust emission areas of Iceland (*16*, *24*) within ~5 hours of being sampled. In contrast, 75% of the samples for which the air masses had a cleaner origin (they did not pass through the boundary layer above the dust emission areas of Iceland) had very low aerosol concentrations. Satellite imagery confirms the presence of dust plumes emanating from sources in southern Iceland close to the location and time for some of the samples collected here (Fig. 1C). A few of the trajectories indicate that potential HLD sources in eastern Greenland might also contribute to the dust loadings. However, examination of the chemical composition of the collected dust particles revealed that the airborne dust we collected always had a chemical composition close to the chemical composition of bulk Icelandic dust or volcanic ash (see section S3 and fig. S7). Furthermore, it has a different chemical signature than those of dust particles collected in other locations at lower latitudes (fig. S7D). Hence, we conclude that the dust we sampled on these flights was predominantly of Icelandic origin.

**Fig. 2:**
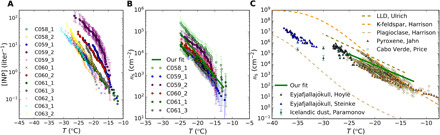
Ice nucleation ability of the Icelandic dust samples. (**A**) INP concentration (per standard liter of atmosphere) for each sample. The INP concentration spectrum of the samples marked with an “x” should be taken as an upper limit, because the INP signal they produced was only slightly above the limit of detection (the corresponding fraction of droplets frozen for each sample is shown in fig. S8). For simplicity, the errors have only been shown for one sample. (**B**) Density of active sites of each sample, plotted with our fit to the data [*n*_s_(*T*) = 10^–0.0337–0.199*T*^, with *T* between −12.5° and −25°C]. Only samples that display an INP concentration substantially above the limit of detection are shown here. Errors in all samples have been shown. (**C**) Icelandic dust density of active site parameterization from this study compared with volcanic ash data from Eyjafjallajökull (*28*, *29*), Icelandic dust (*27*), pyroxene (*35*), laboratory study–based parameterizations for LLD (*4*), K-feldspar and plagioclase (*6*), as well as airborne Saharan dust samples (*33*).

The INP concentration as a function of temperature for each sample was then determined using a cold-stage droplet freezing assay where droplets were placed on top of the Teflon filter (*33*). Figure 2A shows the INP concentration of the collected aerosol samples, while the fraction of droplets frozen at each temperature is presented in fig. S8. The INP concentrations scatter through two orders of magnitude for a given temperature, with some low-activity samples not having an INP signal substantially above the limit of detection. Samples that were observed to have the highest numbers of INP also exhibited the largest dust surface area concentrations (see fig. S9). Samples with aerosol particles above the limit of detection (all of them apart from one also exhibited INP concentrations above the limit of detection) were dominated by dust, with the remainder corresponding mainly to sea spray and carbonaceous particles. Consequently, we assumed that the ice-nucleating ability of the samples is imparted by the presence of dust particles, and so our INP concentration and SEM dust surface area measurements were used to calculate the density of active sites [*n*_s_(*T*)] of the sampled Icelandic dust (Fig. 2B) and derive a parameterization.

The *n*_s_(*T*) parameterization for Icelandic dust is compared with other mineral dust and ash parameterizations and data in Fig. 2C. Icelandic dust shows an activity slightly lower than that reported for LLD in a laboratory study (*4*). However, the values overlap with those reported for airborne desert dust, using the same experimental approach as used here (*33*), although with a shallower slope that results in higher activity above about −17°C. Icelandic dust also has a shallower slope of *n*_s_ versus *T* when compared to pure K-feldspar (*6*), resulting in ice-nucleating activity larger than K-feldspar above about −18°C. The different slope and high activity at higher temperatures suggests that standard K-feldspar may not control the ice-nucleating ability of Icelandic dust, which is consistent with other studies that indicate that the relationship between mineralogy and the ice-nucleating activity of volcanic material is more complex (*27*, *31*) than in low LLDs, where K-feldspar is thought to control its ice-nucleating activity (*6*). In addition, Icelandic soils are not likely to contain major amounts of K-feldspar, but other minerals such as plagioclases and pyroxenes as well as glasses are one of the main components of these soils (*24*, *34*). As shown in Fig. 2C, the ice nucleation ability of plagioclases is orders of magnitude lower than our Icelandic dust samples. Similarly, it was recently shown that glasses of volcanic origin are also very poor at nucleating ice (*31*). In contrast, another recent study indicates that pyroxenes exhibit a similar slope and comparable activity to the airborne Icelandic dust samples (*35*). It is also possible that an ice-active biological component contributes to the ice-nucleating activity of Icelandic dust from glacio-fluvial processes, as was the case in Svalbard (*11*). Unfortunately, further analysis for biological INP or mineralogy was not practical with the small quantities of dust collected using the aircraft sampling system. The ice-nucleating ability of our samples is consistent with that reported for ash from the 2010 eruption of Eyjafjallajökull (*28*, *29*) and surface-sampled glaciogenic silt (*27*). Overall, the airborne Icelandic dust we sampled is a relatively active material and is more active than LLD at temperatures above −17°C but substantially less active at lower temperatures.

### Modeling the emission and transport of Icelandic dust to assess its importance as an INP source

To determine how important Icelandic dust is relative to LLD, we have used a global aerosol model called GLOMAP (*36*). This model has been used previously to represent the global distribution of desert dust and the organic part of sea spray acting as INPs (*5*). This model simulates the emission, transport, and microphysical processing of size-resolved aerosol particles containing several chemical species including dust. The ice-nucleating ability of LLD is quantified in terms of the K-feldspar content (emitted as a fraction of the dust mass and tracked separately in the model) (*5*), which is considered to be the most important ice-nucleating mineral in LLD (*6*). Sea spray INPs are linked to the organic fraction of sea spray aerosol according to a parameterization based on the ice-nucleating ability of ocean surface microlayer samples (*37*). Sea spray was always a minor component of the INP population in this location according to our calculations when compared with desert dust, in agreement with a previous study (*5*). Therefore, we will focus our study on comparing Icelandic dust with LLD.

Previous versions of GLOMAP did not include dust emission sources above 42°N, but here, an Icelandic dust source was added to the existing AEROCOM inventory (*7*) on the 28 days (in 2001) when dust storms were recorded (*15*). These daily emissions were tuned (from the AEROCOM median) to reproduce monthly mean dust mass concentration measurements at Heimaey, an island of the south coast of Iceland (fig. S10) (*14*, *15*). Our tuned yearly emissions (for 2001) of ~5 Tg (0.2% of global emissions) are a factor of 6 lower than that estimated from deposition rates by a previous work (*24*). This underestimation is likely the result of only tuning against southern observations (underestimating the magnitude of northern directed dust plumes) and also because 2001 was a relatively quiescent dust year. Thus, our modeled results most likely represent the lower limit of the Icelandic dust impact. We did this modeling for 2001 because we have already published global INP distributions in this model for the year 2001 (*5*, *37*, *38*). INP concentrations from both Icelandic dust and from LLD have been calculated using a method similar to a previously defined approach (*5*). In the case of LLD, we combined the K-feldspar concentrations simulated by the model for the year 2001 with the most recent K-feldspar parameterization (*6*). Icelandic dust concentrations have been combined with the parameterization of our dust samples shown in Fig. 2C. We make the assumption that the dust we sampled in October 2017 is representative of Icelandic dust in general and its activity would not vary with year or season. All the INP concentrations shown in this study correspond to INP concentration active at ambient temperature, [INP]_ambient_. [INP]_ambient_ is defined as the concentration active at the local temperature of the grid box (*5*). This is in contrast to [INP]*_T_*, which is the INP active at some defined *T*, usually the set point of an INP instrument. An INP’s potential to nucleate ice will only be realized if it is exposed to sufficiently low temperatures; hence, [INP]_ambient_ is a useful measure of where aerosol that have the potential to nucleate ice exist and where the temperatures are low enough for them to do so. Evaluating the global distribution of INP produced by both the LLD and the Icelandic sources allows us to determine whether Icelandic dust is an important source of INP at cloud altitudes relative to LLD.

**Fig. 3:**
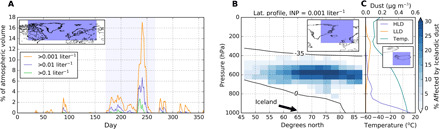
Temporal and altitude distribution of Icelandic dust INPs. (**A**) Daily percentage of atmospheric volume where Icelandic dust INPs dominate over the LLD INPs, leading to three given total INP concentrations. The calculations have been done over the area indicated in the inset map [−100°W, 60°E] and [45°N, 90°N]. The percentages have been calculated over the grid boxes with temperatures in the range of −35° to −12.5°C (we limit this analysis to −12.5°C because this is the limit of our parameterization, and the effect at these temperatures is minor). At temperatures below −25°C, we assume no increase in *n*_s_; hence, we underestimate the INP population from Icelandic dust. The shadowed area corresponds to the summer (days 172 to 266). (**B**) Percentage of grid boxes in the mixed-phase cloud range where Icelandic dust INP concentration dominates over the LLD INP concentration and where the total INP concentration reaches 0.001 liter^−1^ over the summer (days 172 to 266) and over a longitude range of [−40°, 10°]. (**C**) Altitude profiles of the average ambient temperature, Icelandic dust, and LLD mass concentration over Iceland (the averaging area of these profiles corresponds to what can be seen in the inset map).

The model-predicted contribution of INP associated with Icelandic dust over a season is shown in Fig. 3A. The episodic nature of the dust emissions is clear from this plot with INP enhancement, at different concentrations, across the region for several days after modeled storm events in Iceland. Icelandic dust emissions have a high temporal variability because most of the dust is emitted during dust events that occur on average 30 times a year and last up to 2 to 3 days (*15*, *16*). We express the contribution of Icelandic dust INP as the percentage of atmospheric volume at mixed-phase temperatures, where the Icelandic INP had a higher concentration than INP from LLD at certain total [INP]_ambient_. We show a time series for three [INP]_ambient_ concentration thresholds (>0.1, >0.01, and >0.001 liter^−1^). While the threshold INP concentrations required to affect cloud properties are very uncertain (*39*), modeling work indicates that in shallow boundary clouds, concentrations larger than 0.001 liter^−1^ may affect the liquid water path, while concentrations larger than 0.1 or 1 liter^−1^ can largely remove liquid water (*2*, *40*). We do this for a large part of the northern hemisphere (−100°W to 60°E and 45°N to 90°N) (see inset in Fig. 3A) and show that up to 16% of grid boxes across this vast area is dominated by Icelandic dust at 0.001 liter^−1^. There is a strong seasonal dependence, with the simulations indicating that Icelandic dust mostly contributes to the INP population from June to September, but also contributes sporadically through the rest of the year. However, not all the Icelandic dust events lead to a substantial INP concentration events because the concentration of active INPs depends strongly on the ambient temperature and the emitted dust may be deposited before reaching a location in which the temperature is cold enough for it to act as INPs.

In Fig. 3B, we examine the altitude profile of modeled Icelandic dust and at what altitude sufficiently low temperatures and sufficiently high dust loading exist to produce an INP concentration of 0.001 liter^−1^ active at ambient temperature. This plot depicts the whole summer period (days 172 to 266) only for the region around Iceland. One can see that Icelandic dust contributes substantially to the INP population between about 700 and 500 hPa (about 3 to 5.5 km), for latitudes above 50°N, dominating the INP population up to 25% of grid boxes in the mixed-phase regime. The mean mixed-phase temperature range over the summer is also shown. Summer mixed-phase clouds can occur between about 400 (2 km) and 800 hPa (7 km) above Iceland and down to sea level for the high Arctic. Icelandic dust can make a substantial contribution to the INP population in the 500- to 700-hPa range. This is demonstrated by inspecting the dust mass concentration and temperature profiles in Fig. 3C. While the highest concentration of Icelandic dust is found at sea level, sufficiently cold temperatures only exist higher than about 700 hPa to produce an appreciable [INP]_ambient_. Above about 500 hPa, the LLD mass concentration increases as Icelandic dust concentrations continue to decrease and LLD begins to dominate the [INP]_ambient_. Overall, the presence of Icelandic dust increases the probability of cloud glaciation across the region and extends the mixed-phase regime to lower altitudes.

**Fig. 4:**
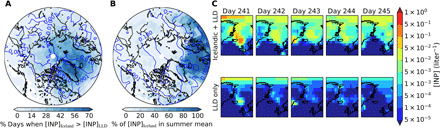
Spatial distribution of Icelandic INPs. (**A**) Fraction of days during the summer (days 172 to 266) in which the INP active at ambient temperature from the Icelandic source dominates over the ones from the LLD source at 550 hPa. The data have been masked to include only days where the INP concentration from the Icelandic source was above 10^−4^ liter^−1^. The contours represent the linear average total INP concentration active at ambient temperature (from both sources) during the days where the Icelandic source dominates, in liter^−1^. (**B**) Fraction of Icelandic INP in the total INP active at ambient temperature (which considers both the Icelandic and the LLD source) at 550 hPa. The contours represent the linear average INP concentration in liter^−1^. (**C**) Example of an Icelandic dust event, which leads to high INP active at ambient temperature concentrations produced by this source at 550 hPa. In the top row, one can see the INP concentration produced by both sources over a period of 5 days, while in the bottom row, only the INP concentration produced by the LLD source has been represented. The temperature range for this event is in between −12.5° and −24.4°C for the areas where the INP concentration active at ambient temperature was above the 10^−4^ liter^−1^ masking. The temperature range for the whole shown area is −4° to −24.5°C.

The spatial distribution of the contribution of Icelandic dust as INP at 550 hPa is shown in Fig. 4. Figure 4A shows a map with the percentage of summer days, where the Icelandic dust was the dominant source of dust INPs active at ambient temperature. At 550 hPa, Icelandic dust contributes more to the INP population than LLD over most of the Arctic Sea, Greenland, some areas of northern Europe, and the North Atlantic for more than one-third of days through the summer. The fraction of days when this happens reaches ~60% of the summer days for areas close to the eastern coast of Greenland and decreases to less than 10% for Alaska, Canada, Siberia, and most latitudes below 50°N. In addition, the contours show that when Iceland was the dominant dust INP source, the INP concentration reached summertime average INP concentrations above 0.001 liter^−1^ for most of the Iceland-dominated areas. Figure 4B shows that Icelandic dust INPs contribute to the summer average total INP concentration (from both the Iceland dust and the LLD) over the North Atlantic and some areas of northern Europe. Over these areas, INP average summertime concentrations reach values above 0.001 liter^−1^. The effect of the Icelandic dust on the total mean INP concentration over the rest of the Arctic is mostly below 10%. However, as shown in Fig. 4A, the Icelandic dust is still the dominant INP type over most of the Arctic for more than 30% of summer days across large parts of the Arctic, because of the sporadic nature of dust concentrations. Note that other sources of INPs have not been added to the model, and they could also contribute to the INP population, but it is clear that Icelandic dust substantially contributes to the INP population in this region relative to LLD.

An example of how Icelandic dust can strongly enhance the INP concentration over a 5-day period is shown in Fig. 4C. The INP concentrations from both the Icelandic dust and LLD (top panel) are orders of magnitude higher than when only LLD sources are represented in the model. The presence of Icelandic dust during the event leads to INP concentrations active at ambient temperature up to 0.5 liter^−1^ over a large area of the North Atlantic compared to less than 0.01 liter^−1^ over a much smaller area if only the LLD sources were considered.

## DISCUSSION

We have measured the ice-nucleating ability of Icelandic dust sampled from FAAM BAe-146 using SEM-EDS dust surface area and INP concentration measurements. We found that the ice-nucleating ability of the sampled Icelandic dust is relatively high, exhibiting comparable values to LLD samples and pure K-feldspar at around −17°C, but with a shallower temperature dependence. Using our results in conjunction with outputs from a global aerosol model (GLOMAP), we calculated global distributions of INPs active at ambient temperature from Icelandic dust storms. These simulations show that Icelandic dust substantially contributes to the INP population active at ambient temperature, often out-competing LLD across large parts of the mid to high latitudes. The greatest contribution of Icelandic dust to the INP population occurs during the summer over large areas of the North Atlantic and the Arctic at altitudes between about 700 and 500 hPa, where mixed-phased clouds are known to occur. At 550 hPa, Icelandic dust was the dominant source of INP for 12 to 60% of the summer days over large areas of the Arctic and North Atlantic. This leads to average summertime INP concentrations active at ambient temperature between 10^−3^ and 10^−2^ liter^−1^, with concentrations of up to 0.5 liter^−1^ over large parts of the Arctic in a modeled event. In addition, our modeled results are likely to represent a lower limit of the impact of Icelandic dust, because the model year (2001) was a low-dust year and also we did not tune to dust emissions moving to the north of Iceland.

The important role of Icelandic dust in the INP population is particularly relevant because Iceland is only one of multiple HLD sources (*9*, *11*, *12*) composing only about 10% of the total HLD dust emissions (*10*). Hence, we anticipate that the combined effect of all the HLD sources could contribute substantially to the INP population at high latitudes. Therefore, the results presented here should be interpreted as a lower limit of the relative importance of HLD for ice nucleation in the Arctic, and further investigation of other HLD is needed to confirm this hypothesis and quantify its contribution. Although Svalbard dust emissions have recently been found to be a potentially substantial source of INPs (*11*), there remain many dust sources that have not been analyzed or added to models, such as Alaska (*13*), Canada (*41*), and Greenland (*42*). The origin, mineralogy, and emission mechanisms of HLD vary strongly from one source to another (*9*, *25*); hence, the HLD ice nucleation ability may also vary substantially. For example, Icelandic dust is made up of glacio-fluvial material, which is of volcanic origin (*24*), whereas dust sources from Svalbard comprise both sediments from river beds and anthropogenic sources from coal mines (*43*) and could contain ice-nucleating biogenic material (*11*). Therefore, it may be necessary to analyze and treat each of these sources separately.

Our conclusion that HLD acts as a substantial source of INPs is relevant for several reasons. Shallow marine supercooled clouds with a high albedo are very sensitive to INP concentration, with INP decreasing the liquid water content and therefore decreasing the shortwave reflectivity of these clouds (*2*, *18*). The reduction in ice fraction, as well as corresponding increase in liquid water, of mixed-phase clouds as a consequence of global warming is an important but highly uncertain negative feedback on climate (*22*), with the uncertainty stemming partly from the oversimplification of mixed-phase cloud-climate feedback processes in climate models (*44*). In addition, mixed-phase clouds over snow and ice exert a longwave radiative heating effect on the surface that can accelerate ice melting (*45*). Because HLD emissions will probably increase under most climate change scenarios due to decreases in snow cover and glacier retreat (*9*), the INP population at mid to high latitudes is likely to increase. Increased INP concentrations would lead to a reduction in supercooled water and a decrease in shortwave reflectivity, potentially counteracting the effect of sea surface warming, to produce a positive climate feedback, which has not yet been considered in climate simulations.

## MATERIALS AND METHODS

### Sampling aerosol particles

Aerosol particles have been sampled on top of filters using the filters inlet system on board of FAAM BAe-146. The system has been characterized previously (*32*). This inlet system can sample accumulation and coarse aerosol particles (up to about 20 μm) on top of two filters at the same time, working under sub-isokinetic conditions. The system tends to enhance coarse aerosol particles, but the enhancement tends to be smaller when sampling with the inlet bypass open (*32*); hence, all the samples were collected with the bypass open. Here, we sample on top of Sartorius polytetrafluoroethylene (PTFE) membrane filters (47 mm diameter with a pore size of 0.45 μm) for the INP concentration drop assay and on top of Whatman Nuclepore polycarbonate track-etched filters (47 mm diameter with a pore size of 0.4 μm) for SEM-EDS analysis. Each filter pair was exposed to air during one or more sampling legs around a particular area, as one can see in Fig. 1A. Sampling was performed at constant altitude when possible, which was most of the cases. The samples were frozen at ~ −18°C on the same day they were collected. During the transport, the samples were kept well below freezing temperatures, never exceeding −8.5°C. The INP concentration analysis was carried out a few days after collection, whereas the SEM-EDS analysis was performed over the following year.

### INP concentration drop assay

Here, we have used the same droplet freezing assay similar to that used previously (*33*). Exposed filters were placed on top of glass slides (Ted Pella cover glass, 48 mm by 60 mm by 0.15 mm), which were placed on top of the cold stage using silicon oil to improve thermal contact. The glass slides were made hydrophobic using Turtle Wax ClearVue Rain repellent solution to prevent frost formation and help the droplet pipetting. About 60 pure water droplets (Milli-Q) with a volume of 2 μl were placed onto the exposed PTFE filters. Then, the droplets were cooled at 1 K min^−1^ within a chamber that was flushed with zero-grade dry nitrogen (0.2 liter min^−1^) to inhibit condensation and frost growth. The freezing process was recorded with a camera while measuring the temperature of the cold stage, which allowed us to obtain the fraction of droplets frozen as a function of temperature, *f*(*T*) (shown in fig. S8). The concentration of INPs was calculated using the equation

$$[\text{INP}](T)=-\text{ln}(1-f(T))\frac{A_{\text{fil}}\;}{V_{a}\alpha }$$ (1)

where *A*_fil_ is the area of the filter exposed to aerosol particles and its value corresponds to 11 cm^2^, *V*_a_ is the volume of sampled air, and α is the area of each droplet in contact with the filter. The value of α was 1.357 mm^2^, calculated from the droplet volume and an assumed contact angle value of 126 ± 3°, under the assumption of a spherical cap geometry. The errors have been calculated using a Monte Carlo simulation, which represents the randomness of the distribution of active sites in the droplet freezing assay, in combination with the uncertainty of the contact angle (*33*).

### SEM analysis

Here, we have applied the approach described previously (*32*) to calculate the size-resolved composition of the aerosol particles on top of polycarbonate filters using SEM-EDS. We used a Tescan VEGA3 XM SEM at the Leeds Electron Microscopy and Spectroscopy Centre (LEMAS) with the AZtecFeature software expansion (Oxford Instruments) for automated particle analysis. The system was operated with an accelerating voltage of 20 keV, a working distance of ~15 mm, and a pixel dwell time of 10 μm. Samples were coated with 30 nm of Ir. Images of the particles on top of filters were used to obtain morphological information of the aerosol particles, while EDS was used to obtain the chemical composition of each individual aerosol particle. The unprocessed data for each x-ray spectra of the analyzed particles were matrix-corrected and normalized by the software (AZtecFeature) to calculate the element weight percentages of the elements present in each particle (*32*). The size-resolved composition, as well as the size distribution of each aerosol sample, was calculated and can be seen in section S2, which allowed us to calculate the dust surface area (*32*). For this analysis, we considered that all particles in the categories Si only, Si rich, Al-Si rich, Ca rich, and Metal rich were dust particles. Particles in the Metal rich category are dominated by either Fe, Cu, Pb, Al, Ti, Zn, or Mn, without major contributions of Si. These particles are metal-containing aerosol particles or metallic oxides from either natural or anthropogenic sources. In our Icelandic aerosol samples, particles in this group contained mainly Fe, with some contributions of Ti and Al, so it was assumed that they are components of the dust. The vast majority of the aerosol particles found in the scanned samples had a chemical composition compatible with mineral dust or volcanic material. The surface area of the dust was calculated by integrating the surface area of the dust particles present in the sample and assuming equivalent circular diameters. A full description of the categories and more information about the setup have been previously shown (*32*).

### Density of active sites calculation and Icelandic dust parameterization

The values of surface area of dust for each of the samples that were scanned using SEM-EDS were used alongside the [INP]*_T_* spectrums to calculate the dust *n*_s_(*T*) spectra of each sample. The calculations were carried out for the selected samples (see fig. S10), where the dust surface areas and INP concentrations were above the limit of detection (C058_1, C059_1, C059_2, C060_2, C061_1, and C061_3), according to the equation

$$n_{s}(T)=\frac{\left[\text{INP}](T\right)}{s}$$ (2)

where *s* is the surface area of the nucleating material (in this case, dust). These calculations are carried out based on the assumption that the ice-nucleating ability of the sample is determined by dust, which is a valid assumption because in the samples shown in Fig. 2, more than 88% of the surface area is constituted by dust particles. The remaining percentage of the surface area was dominated by Na-rich (most likely sea salt) and carbonaceous particles (black carbon, biogenic particles, and artifacts from the filter). The obtained concentrations of sea salt are too small to compete with the dust particles (*46*). In addition, the INP concentrations correlate well with the dust surface areas, as one can see in fig. S9, which also indicates that some component of the dust nucleates ice in these samples. However, we cannot rule out the presence of some ice-active biogenic material associated with the dust particles. In Fig. 2C, one can see the fit that was applied to the selected samples, *n*_s_(*T*) = 10^–0.0337–0.199*T*^, with *T* between −12.5° and −25°C. The fit is comparable with the LLD and K-feldspar parameterizations.

### Global aerosol model

The global aerosol model GLOMAP mode used in this work runs at a horizontal resolution of 2.8° × 2.8° (~60 km^2^ in the high Arctic) with 31 pressure levels from the surface to 10 hPa (*36*). The model represents the atmospheric evolution of six different aerosol species (SO_4_, black carbon, organic carbon, NaCl, dust, and feldspar) distributed in seven lognormal modes (four soluble and three insoluble). Feldspar is emitted as a fraction of the dust mass and tracked separately in the model, but it is treated similarly. The model simulates several aerosol microphysical processes using the ERA-Interim reanalysis fields for the atmospheric dynamics. Dust and feldspar are emitted into the accumulation (~100 to 1000 nm) and coarse (>1000 nm) modes. Freshly emitted dust devoid of soluble materials is thought to be a poor Cloud Condensation Nucleus (CCN) relative to other more hygroscopic aerosol particles. Hence, we emit dust into the insoluble modes, where they cannot serve as CCN and are therefore not subject to wet removal. However, dust rapidly ages in the atmosphere, becoming internally mixed with soluble material, which makes it more hygroscopic and can then more readily serve as CCN. In GLOMAP, dust is aged via interaction with SO_2_ and moves into the soluble modes (i.e., susceptible to wet scavenging); this occurs on a time scale of hours. The mean lifetime of modeled dust is therefore 3 to 4 days (*36*). Because ice nucleation in mixed-phase clouds is thought to be dominated by particles immersed in cloud droplets (*1*), we assume that only the INP in the soluble modes nucleate ice. We tested the impact of including the insoluble modes on the INP population, and the INP concentrations only increase marginally (e.g., the peaks “% of atmospheric volume” in Fig. 3A increase about 1%).

Global dust emissions were taken from the AEROCOM daily dust inventory (*7*). The model is run from 1 January to 31 December 2001 with daily output. The Icelandic dust emissions, which are not typically represented in GLOMAP, were incorporated into the model using dust climatology data from (*15*). Icelandic dust emissions were isolated by running the model with the Icelandic dust emissions and without them and subtracting the outputs. [INP]_ambient_ values were determined using the temperatures from ECMWF (European Centre for Medium-Range Weather Forecasts) reanalysis fields.

## Supplementary Material

aba8137_Movie_S1.mp4

aba8137_SM.pdf

## SUPPLEMENTARY MATERIALS

Supplementary material for this article is available at http://advances.sciencemag.org/cgi/content/full/6/26/eaba8137/DC1
