# Supplementary material for: Iceland is an episodic source of atmospheric ice-nucleating particles relevant for mixed-phase clouds
Source: Sci Adv. 2020 Jun 24;6(26):eaba8137. doi: 10.1126/sciadv.aba8137 (PMC7314534; doi:10.1126/sciadv.aba8137)
Supplement: aba8137_SM.pdf [file aba8137_SM.pdf]

Science Advances
AAAS

advances.sciencemag.org/cgi/content/full/6/26/eaba8137/DC1

Supplementary Materials for

Iceland is an episodic source of atmospheric ice-nucleating particles relevant for mixed-phase clouds

A. Sanchez-Marroquin*, O. Arnalds, K. J. Baustian-Dorsi, J. Browse, P. Dagsson-Waldhauserova, A. D. Harrison, E. C. Maters, K. J. Pringle, J. Vergara-Temprado, I. T. Burke, J. B. McQuaid, K. S. Carslaw, B. J. Murray

*Corresponding author. Email: py15asm@leeds.ac.uk

Published 24 June 2020, *Sci. Adv.* **6**, eaba8137 (2020)
DOI: 10.1126/sciadv.aba8137

The PDF file includes:

Sections S1 to S6
Table S1
Figs. S1 to S10
Legend for movie S1
References

Other Supplementary Material for this manuscript includes the following:

(available at advances.sciencemag.org/cgi/content/full/6/26/eaba8137/DC1)

Movie S1

Section S1. Information about the collected samples

Sample	Day (Oct 2017)	Start time	End time	Mean altitude (m)	Vol. PC (L)	Vol. tef. (L)	PTFE position	Dust ($\mu m^2/cm^3$)	% dust	Mass ($\mu g/m^3$)	INP_{-20} (L^{-1})	Air mass	Mode centre (~µm)
C058_1	2	11:13:00	11:41:45	307 (162)	601	352	Up	5.1	88	16	0.54	Land	6
C058_2	2	11:58:30	12:07:30	328 (183)	316	117	Low	8.6	92	26	0.47†	Land	8
C059_1	2	16:23:45	16:49:30	324 (186)	432	291	Low	157.7	93	297	7.2	Land	3
C059_2	2	16:52:20	17:01:35	242 (154)	185	28	Up	103.4	94	191	20.0	Land	3
C059_3	2	17:16:00	17:37:10 *	1305 (1154)	347	147	Up	Damaged	-	-	0.82†	No land	-
C060_1	4	09:53:30	10:05:30	778 (265)	328	170	Low	6.2	93	13	0.59†	Land	5
C060_2	4	11:22:20	11:29:10	131 (39)	216	101	Low	86.5	99	183	5.2	Land	5
C061_1	4	15:18:10	15:58:45 *	2720 (2044)	343	362	Low	30.2	89	58	1.39	No land	5
C061_2	4	16:54:00	17:05:00	406 (308)	387	115	Up	Damaged	-	-	Damaged	-	-
C061_3	4	17:46:25	18:02:00	253 (170)	468	350	Low	56.5	95	164	1.61	Land	6
C062_1	5	13:43:00	13:53:15	3052 (2482)	103	97	Low	4.2†	28†	48†	0.93†	No land	-
C063_1	5	16:57:15	17:02:25	1924 (1346)	86	73	Up	1.8†	14†	33†	0.67†	No land	-
C063_2	5	17:15:20	17:51:10 *	415 (232)	345	40	Up	1.08†	37†	15	1.36†	No land	-

Table S1. Details of all the pairs of filters analysed during the campaign. Each entry corresponds to the pair of polycarbonate (PC) and Polytetrafluoroethylene (PTFE) filters collected at a particular time. The given days correspond to October 2017. The first given mean altitude corresponds to barometric altitude, while the magnitude in parenthesis corresponds to the radar altitude. The volumes sampled for each filter, as well as the inlet position of the PTFE filter (which is opposite to that of the PC filter in each filter pair) are given. The surface area of dust, calculated by integrating the areas of the aerosol particles in the Si rich, Si only, Al-Si rich, Ca rich and Metal rich as well as the INP concentrations at -20 ^{o}C (from Fig. 2A) are given. An estimation of the mass of the dust present of the sample has been calculated by integrating the volume size-distribution and assuming a density of 2.6 g cm^{-3}. Note that these mass values are likely to be overestimating the atmospheric mass concentrations more than the dust surface area since the sub-isokinetic enhancement of the inlet system is larger at the peak of the volume size-distributions. Using the back trajectories shown in Fig. 1B, the samples have been labelled as those which passed through the boundary layer over the surface of Iceland (Land) and those which did not (No land). An estimation of the centre of the mode of the aerosol surface area size distribution has also been provided when possible. Note that both filters collected during the C061_2 run, as well as the PC filter collected during the C059_3 run were damaged and so they have not been analysed. The symbol * indicates that there was at least one interruption during the sampling to avoid sampling during a turn or altitude change. The symbol † indicates that the quantity is in the limit of detection.

Section S2. Size-resolved composition of Icelandic aerosol samples obtained using SEM

Here we show the surface area size distributions and size-resolved compositional graphs of all the analysed samples. Equivalent circular diameters were used for these calculations. In addition, the SEM derived distributions are compared with the distributions derived from the optical probes on board of the FAAM BAe-146 (the Passive Cavity Aerosol Spectrometer Probe 100-X (PCASP) and Cloud Droplet Probe (CDP)), using the calibration method described previously (*32*) and (*47*). Data from the optical probes is expressed in terms of optical diameter. Note that the diameters of the SEM and optical probes size distributions are different, which could be contributing to the discrepancies in between the instruments. Details on how the size-resolved composition of each aerosol sample is acquired and an interpretation of each compositional category have also been discussed previously (*32*). Note that the size distributions of the samples collected during the C062 and C063 flights are consistent with the limit of detection due to the low number of particles present on the filter. The limit of detection of dust surface area is about 1 $\mu m^2\ cm^{-3}$ for a 400 L sample and about 4 $\mu m^2\ cm^{-3}$ for a 100 L sample.

S2.1 CO58

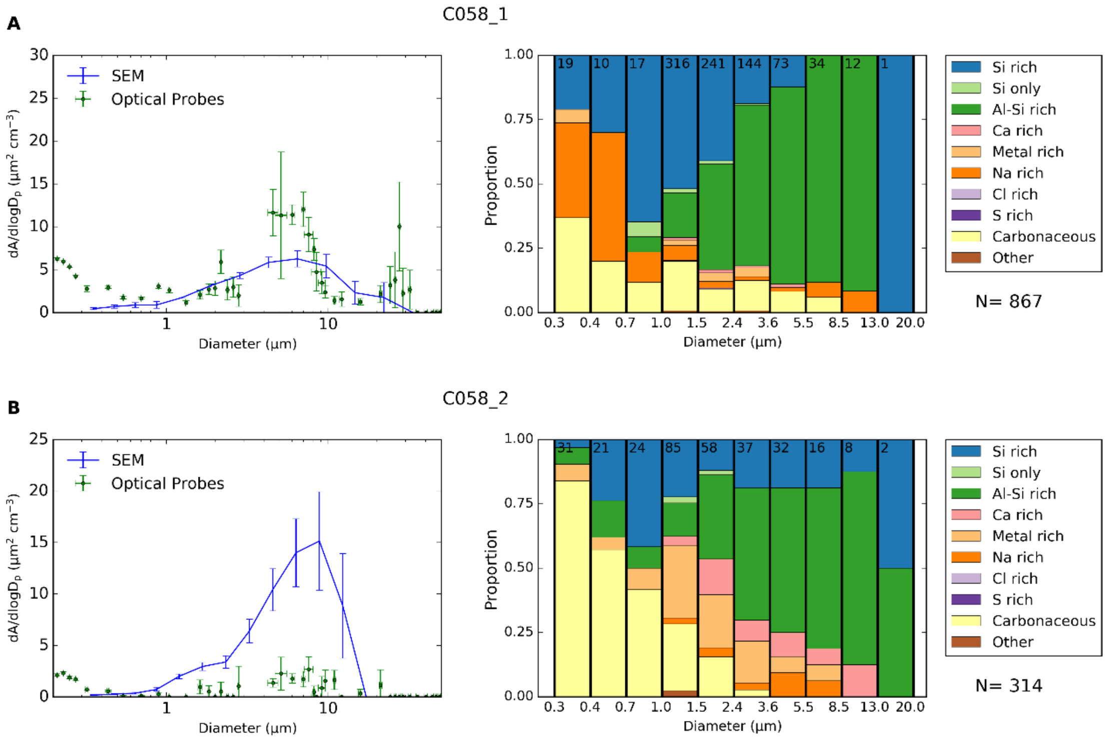

Figure S1. Surface area size distribution and size-resolved composition of the samples collected during the C058 flight. The disagreement in between the optical probes and the SEM counting in the C058_2 sample is probably due to the low number of particles present in the sample.

S2.2 C059

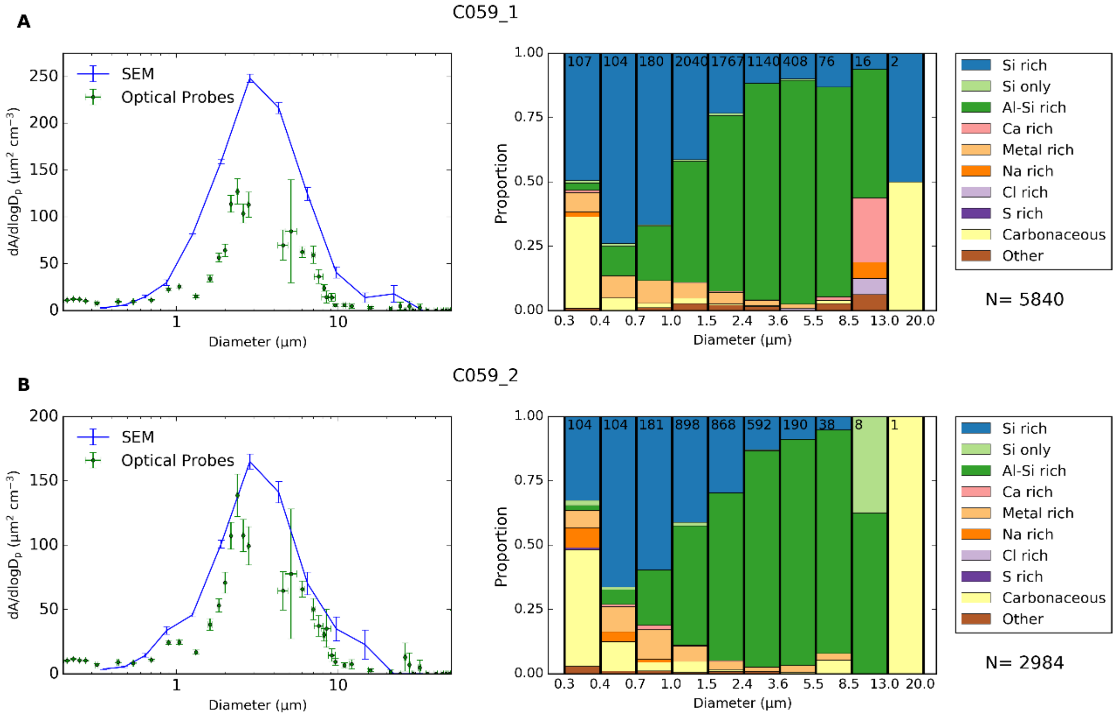

Figure S2. Surface area size distribution and size-resolved composition of the samples collected during the C059 flight.

S2.3 C060

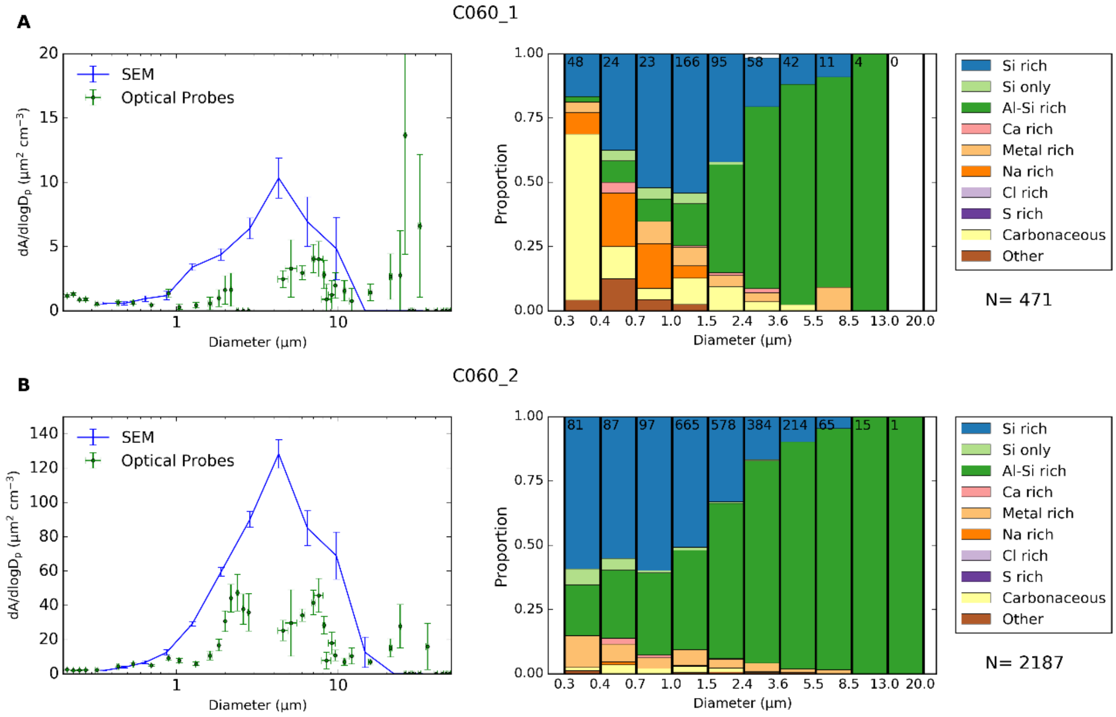

Figure S3. Surface area size distribution and size-resolved composition of the samples collected during the C060 flight.

S2.4 C061

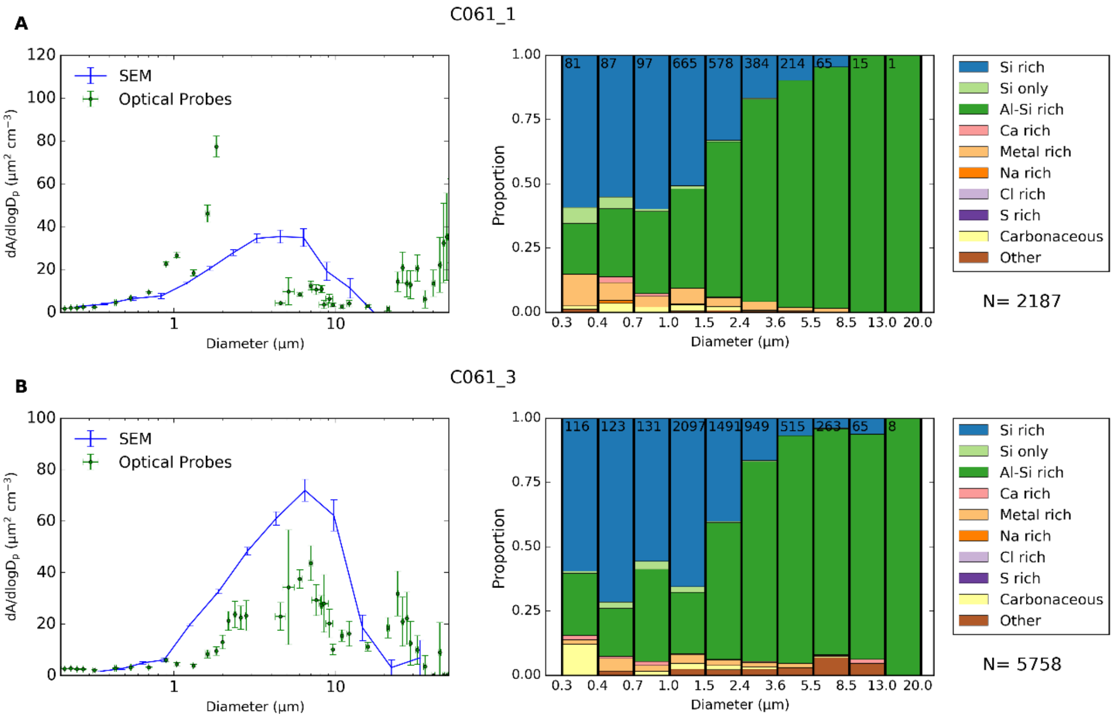

Figure S4. Surface area size distribution and size-resolved composition of the samples collected during the C061 flight. The disagreement in between the optical probes and the SEM counting at ~2 µm could be produced by some artefacts such as water droplets in the PCASP instrument.

S2.5 C062

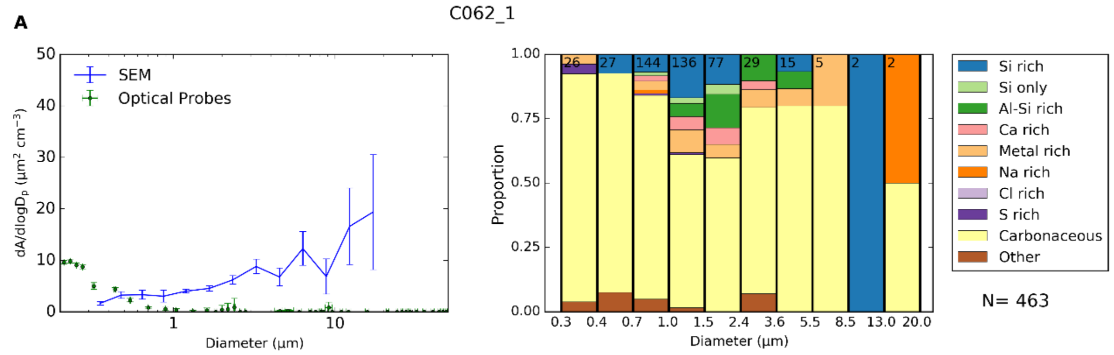

Figure S5. Surface area size distribution and size-resolved composition of the samples collected during the C062 flight. The disagreement in between the optical probes and the SEM counting in the C062_1 sample is probably due to the low number of particles present in the sample present in the filter sample.

S2.6 C063

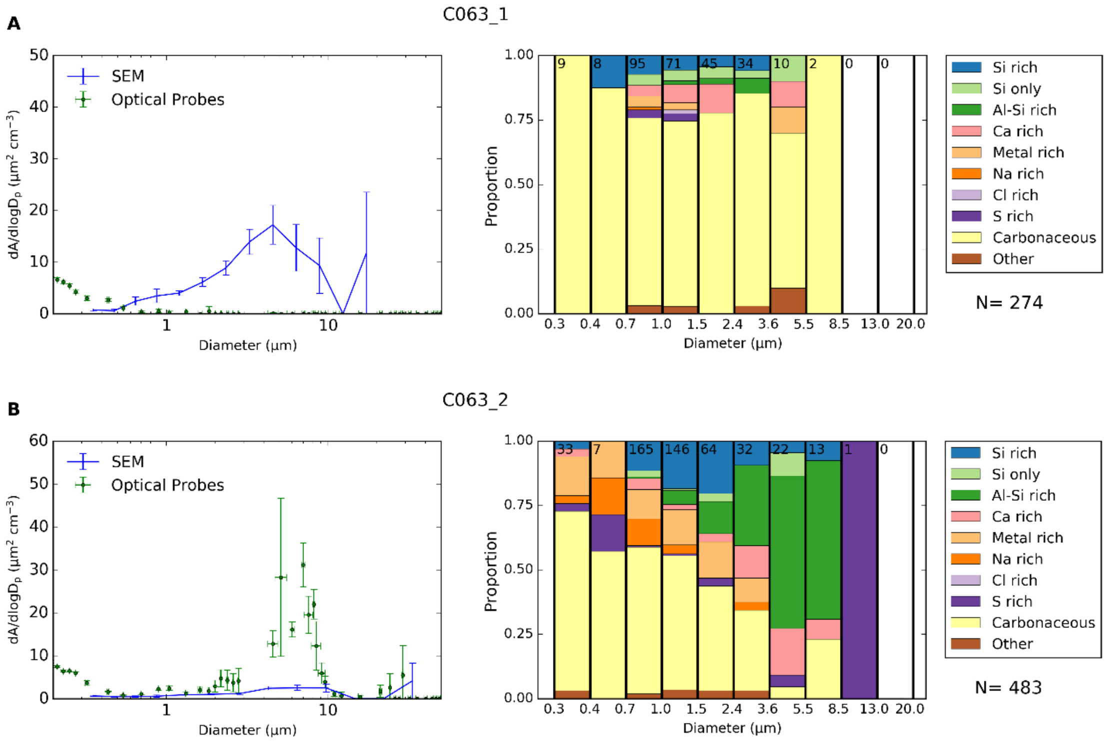

Figure S6. Surface area size distribution and size-resolved composition of the samples collected during the C063 flight. The disagreement in between the optical probes and the SEM counting in the C063_1 sample is probably due to the low number of particles present in the filter sample. In the C063_2, the disagreement could be produced by artefacts in the CDP instrument.

Section S3. Dust chemical composition

Here we show more detailed analysis of the chemical composition of the dust particles collected in this study (all particles in the categories Si rich, Si only, Al-Si rich, Ca rich and Metal rich). Fig. S7A displays the contribution of each element measured for each aerosol sample. The composition of most samples is relatively consistent with the other samples, being more consistent for the most abundant elements (such as Si) and showing a larger scatter for the less abundant elements (such as Ti). The only exception is sample C061_1, which contains a larger fraction of Metal rich (in this case Al rich) particles (Fig. S4). In addition, in Fig. 1B one can see that the air mass where this sample was collected was the only one in the group of samples dominated by dust particles that did not pass through the boundary layer above Iceland. This means that some of the aerosol particles in this sample, particularly those rich in Al, could have a different origin.

Since the composition of the aerosol particles in each dust sample does not show a large sample-to-sample variability, we merged all of the dust sample compositions for further comparison. In Fig. S7B, one can see that the magnitude and range of concentrations of major elements determined by SEM analysis of our Icelandic dust samples is consistent with previous measurements of Icelandic volcanic ash samples and dust events. The Icelandic dust particles contain larger amounts of Ti than dust particles analysed using the same technique and very likely from low-latitude-dust sources collected in different locations such as the UK and Alaska (*32*) or Barbados, as shown in Fig. S7C. Higher concentrations of Ti in Icelandic samples are consistent with their potential origin from Fe-Ti-rich basaltic magmas (*48*). In Fig S7D differences between the chemical composition of the Icelandic dust particles and dust particles collected in the UK, Barbados and Alaska can be seen. Since Icelandic dust particles contain in general less Si and Al but more Fe, Mg, Ca or Ti, most of them appear as a different mode when compared with the dust particles from other sources.

Overall, it is clear that the Icelandic dust particles have a different chemical signature than other dust particles, which have mainly a lower-latitude origin (particularly in the case of the UK and Barbados). In addition, the chemical composition of the dust particles collected in Iceland is close to literature data of the chemical composition of bulk Icelandic dust and volcanic ash. This information supports the conclusions based on the back trajectory analysis in Fig. 1B, which indicate that the dust samples collected during this study have a local (Icelandic) origin.

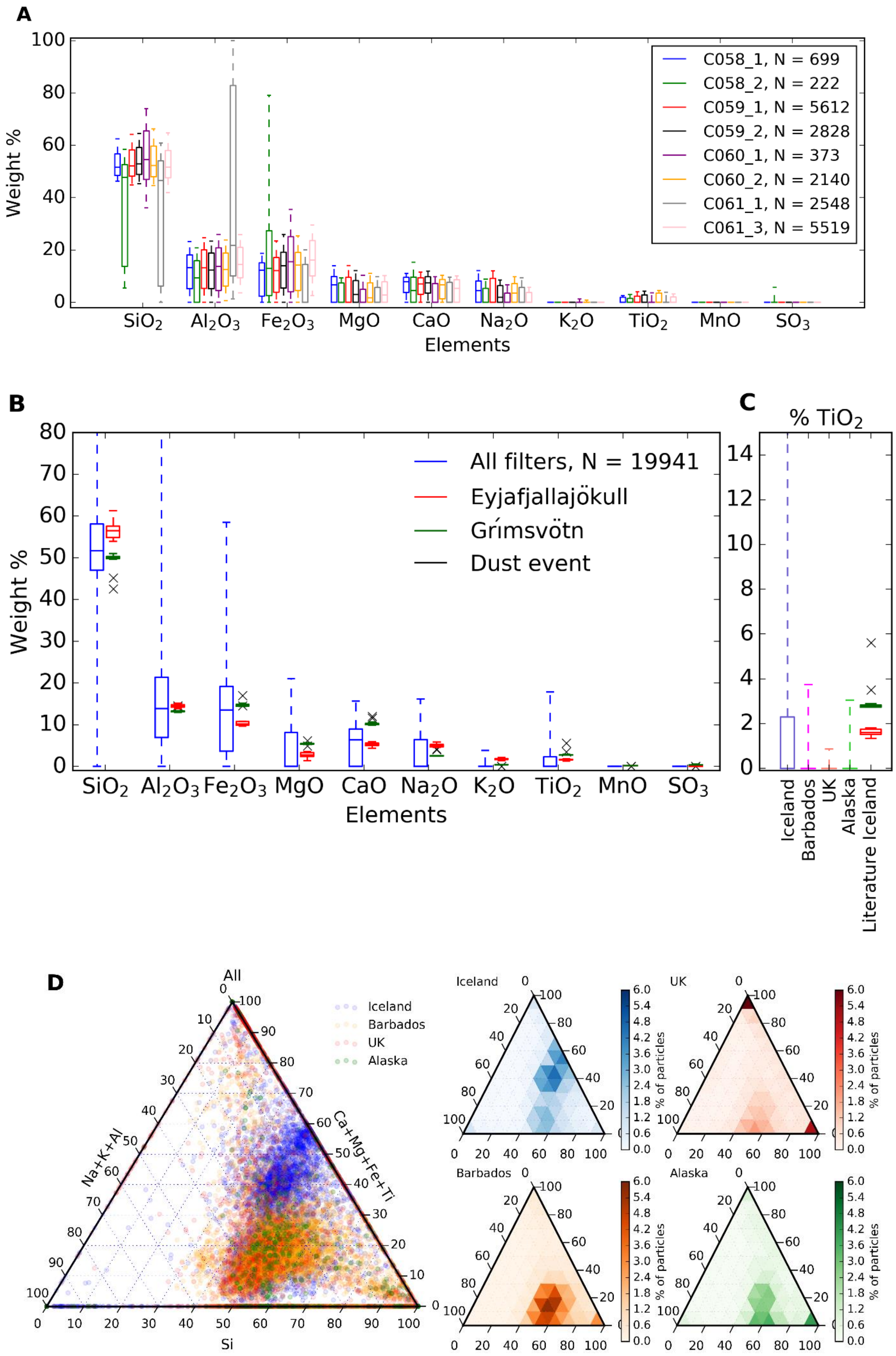
A
Weight %
Elements
C058_1, N = 699
C058_2, N = 222
C059_1, N = 5612
C059_2, N = 2828
C060_1, N = 373
C060_2, N = 2140
C061_1, N = 2548
C061_3, N = 5519
SiO2 Al2O3 Fe2O3 MgO CaO Na2O K2O TiO2 MnO SO3
B
Weight %
Elements
All filters, N = 19941
Eyjafjallajökull
Grímsvötn
Dust event
SiO2 Al2O3 Fe2O3 MgO CaO Na2O K2O TiO2 MnO SO3
C
% TiO2
Iceland
Barbados
UK
Alaska
Literature Iceland
D
All
Iceland
Barbados
UK
Alaska
Na+K+Al
Ca+Mg+Fe+Ti
Si
% of particles

Figure S7. Chemical composition analysis of the Icelandic samples. (a) The boxes represent the median, Q1 and Q3 percentiles of the percentage of the composition of each element in all the dust particles in each sample. The whiskers represent the composition of all particles located in between the median plus and minus two standard deviations. All the SEM analysed samples from this study which have dust surface areas above the limit of detection (all samples apart from C062_1, C063_1 and C063_2) are shown here. Only particles with chemical composition compatible with dust or ash (Si rich, Si only, Al-Si rich, Ca rich and Metal rich) which are not mixed with NaCl have been shown. (b) Composition of all the combined Icelandic aerosol particles from all the samples shown in the previous panel, using the same notation as above. Literature data for bulk composition of different volcanic ash samples from the 2010 Eyjafjallajökull (*49-52*) and 2011 Grímsvötn (*49, 53*) eruptions, as well as two dust events (*54, 55*) have been shown. (c) Weight percentage of the Ti of the collected Icelandic dust particles compared with dust particles from four samples collected in the UK and an Alaskan sample (*32*) and four samples collected in Barbados (Harrison et al., *In prep*) (the whiskers represent the median plus two standard deviations). Note that 31 % of the Icelandic dust particles contained Ti above the limit of detection, while this number was about 3% for the dust particles collected in the UK, Barbados and Alaska. (d) Ternary graphs of the chemical composition of the dust particles shown in the previous panel. The main ternary graph contains the chemical composition of each particle, while the other four graphs contain a heat map with the percentage of dust particles in each sample compositional bin (the axes are the same in all graphs). The chemical composition of each aerosol (used in all the panels shown here) has been recalculated from the weight percentages given by the SEM software, excluding elements that are not Si, Al, Fe, Mg, Ca, Na, K, Ti, Mn and P.

Section S4. Fraction frozen of the Icelandic samples

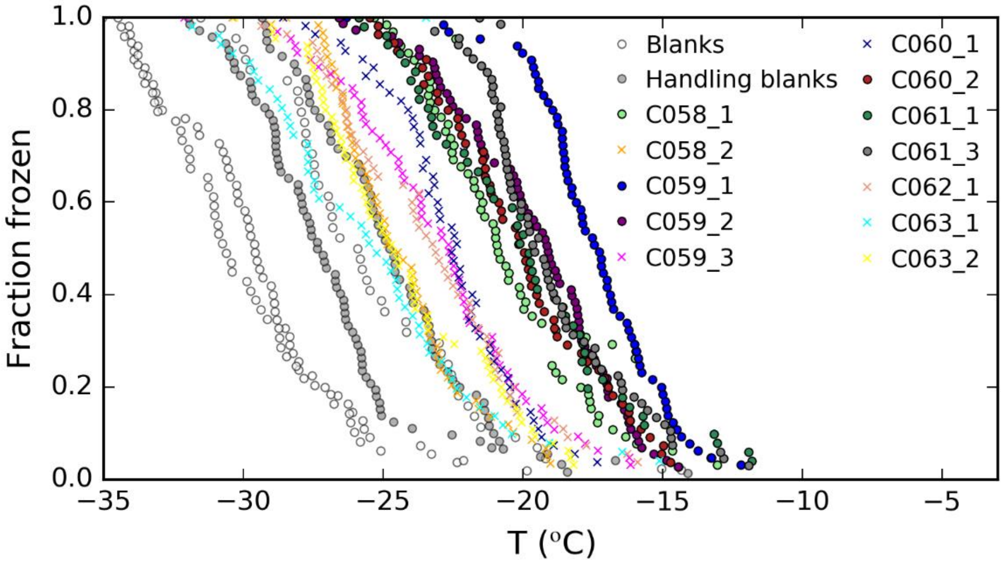

Figure S8. Fraction of droplets frozen at each temperature for each sample. Clean filter blanks as well as handling blanks are also shown. Handling blanks were measured by treating two filters in the same way as filters are treated to collect a sample, but restricting the sampling time to a few second. Samples marked with ‘x’ did not produce a fraction of droplets frozen significantly above the handling blanks so their corresponding INP concentration (in Fig. 2A) are regarded as upper limits of the INP concentration, while samples marked ‘o’ are treated as INP concentration measurements.

Section S5. Correlation in between INP and dust surface area

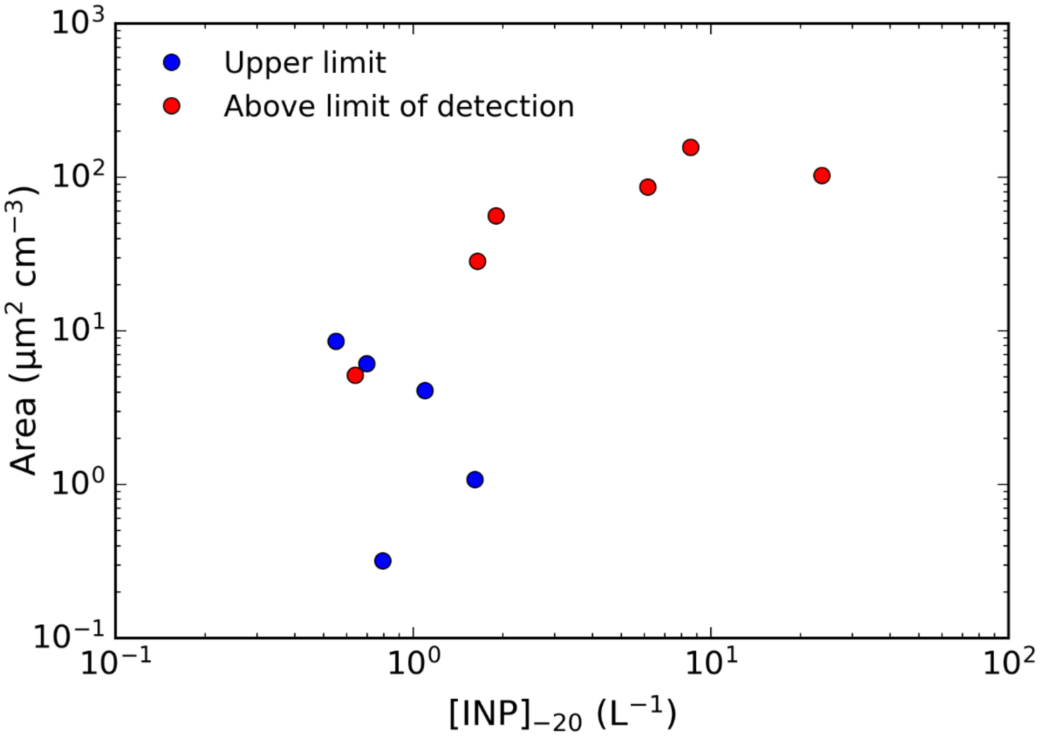

Figure S9. Surface area of dust for each SEM-EDS analysed sample versus the INP concentration at -20 ºC. The samples that did not exhibit a signal clearly above the limit of detection in INP concentration or surface area have been labelled as upper limit.

Section S6. Icelandic dust model inventory

To add Icelandic dust emissions to an existing global inventory (*7*), we added dust plumes to two grid-boxes over Iceland representing the North East and Southern storm regions previously identified (*15*) (Fig S10A). In all, 28 dust storms were added for reported severe events (based in visibility in weather stations) in 2001, which is considered a low dust year (*15*). The timing of these dust events is shown in Fig. S10B. In initial simulations all dust storms were set to the 25th percentile of the global inventory. These initial simulations were then compared to observations at Heimaey in southern Iceland of monthly mean dust concentration (*14*). It was assumed that Icelandic dust was the dominant source of dust at Heimaey. Where the model underestimated monthly mean dust concentrations Icelandic dust emissions were scaled up in that month and where the model overestimated monthly mean dust concentrations Icelandic dust emissions were scaled down. In all model iterations, scaling of daily Icelandic dust emissions was capped at the 75th percentile of the global inventory to prevent unrealistic emissions. This process was repeated until the model was able to reproduce dust concentrations within a reasonable error (-37% bias).

The comparison of our model with both multi-day and monthly mean observations is shown in Fig. S10B and Fig. S10C. Overall we were able to capture the majority of episodic dust enhancements observed at Heimaey (even while tuning to monthly means) with the exception of events where no dust storms were recorded. Our modelled annual mean dust concentration is 3 $\mu g\ m^{-3}$ comparing well to the 4.5 $\mu g\ m^{-3}$ observed and a significant improvement on the baseline model (which did not include the Icelandic dust emissions), which reported 0.09 $\mu g\ m^{-3}$.

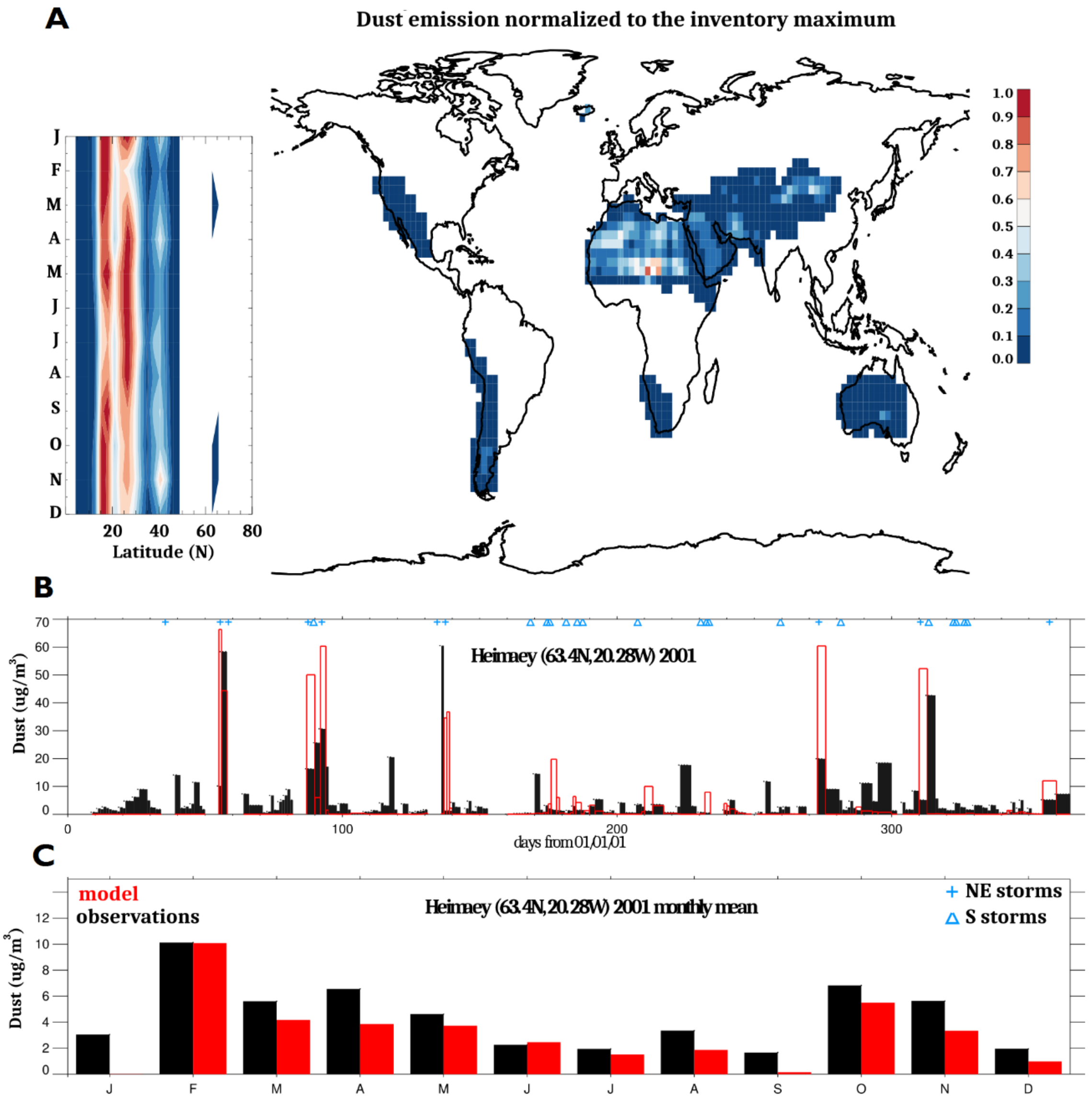

Figure S10: Description of the modelled Icelandic dust emissions. (A) Global dust emissions compared to tuned Icelandic emissions. Here, dust emission rates have been normalised to the inventory maximum. The map indicates the total annual emission (~5 Tg from Iceland) while normalized monthly emissions over latitude are shown in the contour pot. (B) Observed (black) and modelled (red) atmospheric dust concentration at Heimaey, in South Iceland, taken as multi-day (3-5) means (*14*). The individual dust storms are indicated in the top plot by the blue symbols (+ for NE storms, triangle for Southern storms). Tuned emissions were only included on days with reported dust events. (C) Monthly means of the observed and modelled dust concentrations. The model run shown includes our tuned Icelandic dust emission inventory.

Supplementary Materials

Movie S1. Daily $[INP]_{ambient}$ concentration. The data is coloured in red when LLD is the dominating INP source and in blue when Icelandic dust is the dominating INP source.

REFERENCES AND NOTES

1. Z. A. Kanji, L. A. Ladino, H. Wex, Y. Boose, M. Burkert-Kohn, D. J. Cziczo, M. Krämer, Overview of ice nucleating particles. *Meteorol. Monogr.* **58**, 1.1–1.33 (2017).

2. J. Vergara-Temprado, A. K. Miltenberger, K. Furtado, D. P. Grosvenor, B. J. Shipway, A. A. Hill, J. M. Wilkinson, P. R. Field, B. J. Murray, K. S. Carslaw, Strong control of Southern Ocean cloud reflectivity by ice-nucleating particles. *Proc. Natl. Acad. Sci. U.S.A.* **115**, 2687–2692 (2018).

3. Y. Boose, A. Welti, J. Atkinson, F. Ramelli, A. Danielczok, H. G. Bingemer, M. Plötze, B. Sierau, Z. A. Kanji, U. Lohmann, Heterogeneous ice nucleation on dust particles sourced from nine deserts worldwide—Part 1: Immersion freezing. *Atmos. Chem. Phys.* **16**, 15075–15095 (2016).

4. R. Ullrich, C. Hoose, O. Möhler, M. Niemand, R. Wagner, K. Höhler, N. Hiranuma, H. Saathoff, T. Leisner, A new ice nucleation active site parameterization for desert dust and soot. *J. Atmos. Sci.* **74**, 699–717 (2017).

5. J. Vergara-Temprado, B. J. Murray, T. W. Wilson, D. O'Sullivan, J. Browse, K. J. Pringle, K. Ardon-Dryer, A. K. Bertram, S. M. Burrows, D. Ceburnis, P. J. DeMott, R. H. Mason, C. D. O'Dowd, M. Rinaldi, K. S. Carslaw, Contribution of feldspar and marine organic aerosols to global ice nucleating particle concentrations. *Atmos. Chem. Phys.* **17**, 3637–3658 (2017).

6. A. D. Harrison, K. Lever, A. Sanchez-Marroquin, M. A. Holden, T. F. Whale, M. D. Tarn, J. B. McQuaid, B. J. Murray, The ice-nucleating ability of quartz immersed in water and its atmospheric importance compared to K-feldspar. *Atmos. Chem. Phys.* **19**, 11343–11361 (2019).

7. N. Huneeus, M. Schulz, Y. Balkanski, J. Griesfeller, J. Prospero, S. Kinne, S. Bauer, O. Boucher, M. Chin, F. Dentener, T. Diehl, R. Easter, D. Fillmore, S. Ghan, P. Ginoux, A. Grini, L. Horowitz, D. Koch, M. C. Krol, W. Landing, X. Liu, N. Mahowald, R. Miller, J. J. Morcrette, G. Myhre, J. Penner, J. Perlwitz, P. Stier, T. Takemura, C. S. Zender, Global dust model intercomparison in AeroCom phase I. *Atmos. Chem. Phys.* **11**, 7781–7816 (2011).

8. P. Ginoux, J. M. Prospero, T. E. Gill, N. C. Hsu, M. Zhao, Global-scale attribution of anthropogenic and natural dust sources and their emission rates based on MODIS Deep Blue aerosol products. *Rev. Geophys.* **50**, RG3005 (2012).

9. J. E. Bullard, M. Baddock, T. Bradwell, J. Crusius, E. Darlington, D. Gaiero, S. Gassó, G. Gisladottir, R. Hodgkins, R. McCulloch, C. McKenna-Neuman, T. Mockford, H. Stewart, T. Thorsteinsson, High-latitude dust in the Earth system. *Rev. Geophys.* **54**, 447–485 (2016).

10. C. D. Groot Zwaaftink, H. Grythe, H. Skov, A. Stohl, Substantial contribution of northern high-latitude sources to mineral dust in the Arctic. *J. Geophys. Res. Atmos.* **121**, 13678-13697 (2016).

11. Y. Tobo, K. Adachi, P. J. DeMott, T. C. J. Hill, D. S. Hamilton, N. M. Mahowald, N. Nagatsuka, S. Ohata, J. Uetake, Y. Kondo, M. Koike, Glacially sourced dust as a potentially significant source of ice nucleating particles. *Nat. Geosci.* **12**, 253−258 (2019).

12. D. S. Hamilton, R. A. Scanza, Y. Feng, J. Guinness, J. F. Kok, L. L. Li, X. H. Liu, S. D. Rathod, J. S. Wan, M. X. Wu, N. M. Mahowald, Improved methodologies for Earth system modelling of atmospheric soluble iron and observation comparisons using the Mechanism of Intermediate complexity for Modelling Iron (MIMI v1.0). *Geosci. Model Dev.* **12**, 3835–3862 (2019).

13. J. Crusius, A. W. Schroth, S. Gasso, C. M. Moy, R. C. Levy, M. Gatica, Glacial flour dust storms in the Gulf of Alaska: Hydrologic and meteorological controls and their importance as a source of bioavailable iron. *Geophys. Res. Lett.* **38**, 1–5 (2011).

14. J. M. Prospero, J. E. Bullard, R. Hodgkins, High-latitude dust over the North Atlantic: Inputs from Icelandic proglacial dust storms. *Science* **335**, 1078–1082 (2012).

15. P. Dagsson-Waldhauserova, O. Arnalds, H. Olafsson, Long-term variability of dust events in Iceland (1949–2011). *Atmos. Chem. Phys.* **14**, 13411–13422 (2014).

16. C. D. Groot Zwaaftink, Ó. Arnalds, P. Dagsson-Waldhauserova, S. Eckhardt, J. M. Prospero, A. Stohl, Temporal and spatial variability of Icelandic dust emissions and atmospheric transport. *Atmos. Chem. Phys.* **17**, 10865–10878 (2017).

17. P. Dagsson-Waldhauserova, O. Arnalds, H. Olafsson, Long-term dust aerosol production from natural sources in Iceland. *J. Air Waste Manag. Assoc.* **67**, 173–181 (2017).

18. U. Lohmann, K. Diehl, Sensitivity studies of the importance of dust ice nuclei for the indirect aerosol effect on stratiform mixed-phase clouds. *J. Atmos. Sci.* **63**, 968–982 (2006).

19. S. Fu, X. Deng, M. D. Shupe, H. Xue, A modelling study of the continuous ice formation in an autumnal Arctic mixed-phase cloud case. *Atmos. Res.* **228**, 77–85 (2019).

20. H. Morrison, G. de Boer, G. Feingold, J. Harrington, M. D. Shupe, K. Sulia, Resilience of persistent Arctic mixed-phase clouds. *Nat. Geosci.* **5**, 11–17 (2012).

21. R. Bennartz, M. D. Shupe, D. D. Turner, V. P. Walden, K. Steffen, C. J. Cox, M. S. Kulie, N. B. Miller, C. Pettersen, July 2012 Greenland melt extent enhanced by low-level liquid clouds. *Nature* **496**, 83–86 (2013).

22. I. Tan, T. Storelvmo, M. D. Zelinka, Observational constraints on mixed-phase clouds imply higher climate sensitivity. *Science* **352**, 224–227 (2016).

23. O. Arnalds, Volcanic soils of Iceland. *Catena* **56**, 3–20 (2004).

24. O. Arnalds, P. Dagsson-Waldhauserova, H. Olafsson, The Icelandic volcanic aeolian environment: Processes and impacts—A review. *Aeolian Res.* **20**, 176–195 (2016).

25. B. Moroni, O. Arnalds, P. Dagsson-Waldhauserová, S. Crocchianti, R. Vivani, D. Cappelletti, Mineralogical and chemical records of icelandic dust sources upon Ny-Ålesund (Svalbard Islands). *Front. Earth Sci.* **6**, 187 (2018).

26. P. Dagsson-Waldhauserova, J. B. Renard, H. Olafsson, D. Vignelles, G. Berthet, N. Verdier, V. Duverger, Vertical distribution of aerosols in dust storms during the Arctic winter. *Sci. Rep.* **9**, 16122 (2019).

27. M. Paramonov, R. O. David, R. Kretzschmar, Z. A. Kanji, A laboratory investigation of the ice nucleation efficiency of three types of mineral and soil dust. *Atmos. Chem. Phys.* **18**, 16515–16536 (2018).

28. C. R. Hoyle, V. Pinti, A. Welti, B. Zobrist, C. Marcolli, B. Luo, Á. Höskuldsson, H. B. Mattsson, O. Stetzer, T. Thorsteinsson, G. Larsen, T. Peter, Ice nucleation properties of volcanic ash from Eyjafjallajökull. *Atmos. Chem. Phys.* **11**, 9911–9926 (2011).

29. I. Steinke, O. Möhler, A. Kiselev, M. Niemand, H. Saathoff, M. Schnaiter, J. Skrotzki, C. Hoose, T. Leisner, Ice nucleation properties of fine ash particles from the Eyjafjallajökull eruption in April 2010. *Atmos. Chem. Phys.* **11**, 12945–12958 (2011).

30. T. P. Mangan, J. D. Atkinson, J. W. Neuberg, D. O'Sullivan, T. W. Wilson, T. F. Whale, L. Neve, N. S. Umo, T. L. Malkin, B. J. Murray, Heterogeneous ice nucleation by soufriere hills volcanic ash immersed in water droplets. *PLOS ONE* **12**, e0169720 (2017).

31. E. C. Maters, D. B. Dingwell, C. Cimarelli, D. Müller, T. F. Whale, B. J. Murray, The importance of crystalline phases in ice nucleation by volcanic ash. *Atmos. Chem. Phys.* **19**, 5451–5465 (2019).

32. A. Sanchez-Marroquin, D. H. P. Hedges, M. Hiscock, S. T. Parker, P. D. Rosenberg, J. Trembath, R. Walshaw, I. T. Burke, J. B. McQuaid, B. J. Murray, Characterisation of the filter inlet system on the FAAM BAe-146 research aircraft and its use for size-resolved aerosol composition measurements. *Atmos. Meas. Tech.* **12**, 5741–5763 (2019).

33. H. C. Price, K. J. Baustian, J. B. McQuaid, A. Blyth, K. N. Bower, T. Choularton, R. J. Cotton, Z. Cui, P. R. Field, M. Gallagher, R. Hawker, A. Merrington, A. Miltenberger, R. R. Neely III, S. T. Parker, P. D. Rosenberg, J. W. Taylor, J. Trembath, J. Vergara-Temprado, T. F. Whale, T. W. Wilson, G. Young, B. J. Murray, Atmospheric ice-nucleating particles in the dusty tropical atlantic. *J. Geophys. Res. Atmos.* **123**, 2175–2193 (2018).

34. P. Dagsson-Waldhauserova, "Variability, origin and physical characteristics of dust aerosol in Iceland," thesis, University of Iceland (2014).

35. L. G. Jahn, W. D. Fahy, D. B. Williams, R. C. Sullivan, Role of feldspar and pyroxene minerals in the ice nucleating ability of three volcanic ashes. *ACS Earth Space Chem.* **3**, 626−636 (2019).

36. G. W. Mann, K. S. Carslaw, D. V. Spracklen, D. A. Ridley, P. T. Manktelow, M. P. Chipperfield, S. J. Pickering, C. E. Johnson, Description and evaluation of GLOMAP-mode: A modal global aerosol microphysics model for the UKCA composition-climate model. *Geosci. Model Dev.* **3**, 519–551 (2010).

37. T. W. Wilson, L. A. Ladino, P. A. Alpert, M. N. Breckels, I. M. Brooks, J. Browse, S. M. Burrows, K. S. Carslaw, J. A. Huffman, C. Judd, W. P. Kilthau, R. H. Mason, G. McFiggans, L. A. Miller, J. J. Nájera, E. Polishchuk, S. Rae, C. L. Schiller, M. Si, J. Vergara Temprado, T. F. Whale, J. P. S. Wong, O. Wurl, J. D. Yakobi-Hancock, J. P. D. Abbatt, J. Y. Aller, A. K. Bertram, D. A. Knopf, B. J. Murray, A marine biogenic source of atmospheric ice-nucleating particles. *Nature* **525**, 234–238 (2015).

38. J. D. Atkinson, B. J. Murray, M. T. Woodhouse, T. F. Whale, K. J. Baustian, K. S. Carslaw, S. Dobbie, D. O'Sullivan, T. L. Malkin, The importance of feldspar for ice nucleation by mineral dust in mixed-phase clouds. *Nature* **498**, 355–358 (2013).

39. M. D. Petters, T. P. Wright, Revisiting ice nucleation from precipitation samples. *Geophys. Res. Lett.* **42**, 8758–8766 (2015).

40. R. G. Stevens, K. Loewe, C. Dearden, A. Dimitrelos, A. Possner, G. K. Eirund, T. Raatikainen, A. A. Hill, B. J. Shipway, J. Wilkinson, S. Romakkaniemi, J. Tonttila, A. Laaksonen, H. Korhonen, P. Connolly, U. Lohmann, C. Hoose, A. M. L. Ekman, K. S. Carslaw, P. R. Field, A model intercomparison of CCN-limited tenuous clouds in the high Arctic. *Atmos. Chem. Phys.* **18**, 11041–11071 (2018).

41. W. G. Nickling, Eolian sediment transport during dust storms: Slims River Valley, Yukon Territory. *Can. J. Earth Sci.* **15**, 1069–1084 (1978).

42. J. E. Bullard, T. Mockford, Seasonal and decadal variability of dust observations in the Kangerlussuaq area, west Greenland. *Arct. Antarct. Alp. Res.* **50**, 1 (2018).

43. A. Dörnbrack, I. S. Stachlewska, C. Ritter, R. Neuber, Aerosol distribution around Svalbard during intense easterly winds. *Atmos. Chem. Phys.* **10**, 1473–1490 (2010).

44. D. T. McCoy, D. L. Hartmann, M. D. Zelinka, in *Mixed-Phase Clouds,* C. Andronache, Ed. (Elsevier, 2018), pp. 215–236.

45. A. Solomon, M. D. Shupe, N. B. Miller, Cloud–atmospheric boundary layer–surface interactions on the greenland ice sheet during the july 2012 extreme melt event. *J. Clim.* **30**, 3237–3252 (2017).

46. P. J. DeMott, T. C. J. Hill, C. S. McCluskey, K. A. Prather, D. B. Collins, R. C. Sullivan, M. J. Ruppel, R. H. Mason, V. E. Irish, T. Lee, C. Y. Hwang, T. S. Rhee, J. R. Snider, G. R. McMeeking, S. Dhaniyala, E. R. Lewis, J. J. B. Wentzell, J. Abbatt, C. Lee, C. M. Sultana, A. P. Ault, J. L. Axson, M. D. Martinez, I. Venero, G. Santos-Figueroa, M. D. Stokes, G. B. Deane, O. L. Mayol-Bracero, V. H. Grassian, T. H. Bertram, A. K. Bertram, B. F. Moffett, G. D. Franc, Sea spray aerosol as a unique source of ice nucleating particles. *Proc. Natl. Acad. Sci. U.S.A.* **113**, 5797–5803 (2016).

47. P. D. Rosenberg, A. R. Dean, P. I. Williams, J. R. Dorsey, A. Minikin, M. A. Pickering, A. Petzold, Particle sizing calibration with refractive index correction for light scattering optical particle counters and impacts upon PCASP and CDP data collected during the Fennec campaign. *Atmos. Meas. Tech.* **5**, 1147–1163 (2012).

48. P. Nikkola, G. H. Gudfinnsson, E. Bali, O. T. Ramo, T. Fusswinkel, T. Thordarson, Signature of deep mantle melting in South Iceland olivine. *Contrib. Mineral. Petrol.* **174**, 1–19 (2019).

49. C. J. Horwell, P. J. Baxter, S. E. Hillman, J. A. Calkins, D. E. Damby, P. Delmelle, K. Donaldson, C. Dunster, B. Fubini, F. J. Kelly, J. S. Le Blond, K. J. Livi, F. Murphy, C. Nattrass, S. Sweeney, T. D. Tetley, T. Thordarson, M. Tomatis, Physicochemical and toxicological profiling of ash from the 2010 and 2011 eruptions of Eyjafjallajokull and Grimsvotn volcanoes, Iceland using a rapid respiratory hazard assessment protocol. *Environ. Res.* **127**, 63–73 (2013).

50. O. Sigmarsson, I. Vlastelic, R. Andreasen, I. Bindeman, J. L. Devidal, S. Moune, J. K. Keiding, G. Larsen, A. Höskuldsson, T. Thordarson, Remobilization of silicic intrusion by mafic magmas during the 2010 Eyjafjallajökull eruption. *Solid Earth* **2**, 271–281 (2011).

51. M. Paque, M. Detienne, E. C. Maters, P. Delmelle, Smectites and zeolites in ash from the 2010 summit eruption of Eyjafjallajökull volcano, Iceland. *BVol* **78**, (2016).

52. E. C. Maters, P. Delmelle, H. P. Gunnlaugsson, Controls on iron mobilisation from volcanic ash at low pH: Insights from dissolution experiments and Mössbauer spectroscopy. *Chem. Geol.* **449**, 73–81 (2017).

53. J. Olsson, S. L. S. Stipp, K. N. Dalby, S. R. Gislason, Rapid release of metal salts and nutrients from the 2011 Grímsvötn, Iceland volcanic ash. *Geochim. Cosmochim. Acta* **123**, 134–149 (2013).

54. P. Dagsson-Waldhauserova, O. Arnalds, H. Ólafsson, L. Skrabalova, G. Sigurðardóttir, M. Branis, J. Hladil, R. Skala, T. Navratil, L. Chadimova, S. Löwis, T. Thorsteinsson, H. Carlsen, I. Jónsdóttir, Physical properties of suspended dust during moist and low wind conditions in Iceland. *Iceland. Agr. Sci.* **27**, 25–39 (2014).

55. P. Dagsson-Waldhauserova, O. Arnalds, H. Olafsson, J. Hladil, R. Skala, T. Navratil, L. Chadimova, O. Meinander, Snow–Dust Storm: Unique case study from Iceland, March 6–7, 2013. *Aeolian Res.* 16, 69–74 (2015).
